# Detection of unknown primary tumor in patients presented with brain metastasis by F-18 fluorodeoxyglucose positron emission tomography/computed tomography

**DOI:** 10.2217/cns-2017-0018

**Published:** 2018-04-30

**Authors:** Zehra Pınar Koç, Pelin Özcan Kara, Ahmet Dağtekin

**Affiliations:** 1 Nuclear Medicine Department, Medical Faculty, Mersin University, Mersin, Turkey; 2 Neurosurgery Department, Medical Faculty, Mersin University, Mersin, Turkey

**Keywords:** brain, fluorodeoxyglucose, metastasis, PET, unknown primary

## Abstract

**Aim:**

F-18 fluorodeoxyglucose (FDG) PET/CT has several advantages in diagnosis of cancer of unknown primary with reported incremental diagnostic value. In this study, we evaluated the patients who were presented with multiple brain metastasis and unknown primary tumor.

**Materials & methods:**

31 patients (17 males, 14 females; mean: 56.1 ± 14.22 years old) with diagnosis of brain metastasis according to histopathology and/or MRI were included into this retrospective study.

**Results:**

The patients presented with hypermetabolic (n = 17; mean SUVmax: 11.6 ± 6.9) or hypometabolic brain lesions with additional different metastatic sites in 13 patients (mean SUVmax: 9.03 ± 4.02). The primary tumor was determined by FDG PET/CT in 20/26 patients (77%) (lung [n = 6], primary brain [n = 9], renal cell carcinoma [n = 2], skin [n = 1], breast [n = 1] and neuroendocrine tumor [n = 1]).

**Conclusion:**

New generation multislice scanners may provide higher detection ratios. The detection rate of FDG PET/CT might be higher than previously reported according to this study.

**Summary points**

- The F-18 fluorodeoxyglucose (FDG) PET/CT is the most suitable and accurate imaging modality for the identification of the unknown primary tumor of the patients with metastatic brain disease.
- This imaging modality provides an overview of the body as a whole body imaging modality.
- The superiority of FDG PET/CT in cancer imaging and staging is well established.
- Additionally, this modality provides an accurate biopsy site and changes the patient's management. F-18 FDG PET/CT may be a preferred method for the identification of the primary tumor as a single first-line modality.
- The second intervention may be decided by the information provided by the PET/CT results.
- Although most of the patients with unknown primary metastatic brain disease rise from lung cancer, there may be unexpected metastatic tumors all over the body.
- Thus, the imaging modality in this workup must include whole body.

Cancer of unknown primary tumors (CUP) is defined as known metastatic involvement of an organ without definite primary site despite detailed research [1]. CUP is also a diagnostic and therapeutic challenge and true diagnosis results in better survival [2]. However, primary site may be achieved in only approximately a third of the patients by conventional radiological approach [3]. However, F-18FDG PET/CT has high diagnostic accuracy and significantly higher identification rates according to recent studies [1].

Additionally, CUP is a heterogeneous group of patients with different presentations such as cervical lymph nodes, brain metastases, bone metastases or liver metastases. Classification of these cases according to their first presentation might achieve true analysis of the diagnostic efficacy of the diagnostic modalities. There are a limited number of series about the patients with unknown primary tumors with brain metastases as first presentation and some case reports in the literature.

In two series related to the patients who presented with brain metastasis with CUP, the imaging with PET has been found to be a sensitive tool [4,5]. Case reports also usually point out that PET/CT might show the primary tumor in most of the cases [6]. The diagnostic accuracy of PET/CT is definitely superior to PET alone and additional diagnostic parameters are added like multislice CT to new generation of PET scanners. Thus, diagnostic power of F-18 FDG PET/CT in this group of patients might be significantly increased. The aim of this study is to investigate the diagnostic efficiency of F-18 FDG PET/CT in detection of primary tumor in patients who presented with brain metastasis as the first presentation.

## Materials & methods

### Patients

31 patients (17 M, 14F; mean: 56.1 ± 14.2 years old) with diagnosis of brain metastasis according to histopathology and/or MRI were included into this retrospective study. The time between first imaging findings of the brain region was within 1 month and in case of brain operation, the time was at least 1 month after the surgical procedure. All of the patients were informed about the imaging procedure and informed consents were obtained. The patients were included in case of metastatic brain disease (concluded by imaging findings or histopathological results or both) without any knowledge of the primary tumors by other imaging findings or histopathological results and excluded in case of previously defined primary tumor or suspicion of a systemic benign disease as an explanation like tuberculosis, presence of pregnancy or age <18 years. No other imaging modality was implicated in the patients other than PET/CT in order to identify the primary site before the PET/CT examination. The further imaging studies were decided according to the results of PET/CT. The ethical approval of the study was obtained from the local ethics committee.

### Imaging procedure

The patients included in this study necessarily underwent PET/CT examination. After fasting for at least 4 h, intravenous injection of approximately 10-mCi (370-mBq) F-18 FDG injections was performed with additional saline flush. PET/CT imaging was performed by an integrated scanner (Discovery 610, PET/CT scanner, General Electric Company, MA, USA). CT scan prior to PET imaging was performed without intravenous contrast administration with 130 kV, 50-mAs, a pitch of 1.5, a section thickness of 5 mm and a field of view of 70 cm. PET imaging was performed just after the CT scan from vertex to foot with 3 min per bed in a 3D acquisition mode.

### Image interpretation

The PET/CT images were investigated by an experienced nuclear medicine physician independently. The decision of final diagnosis was decided according to the histopathological results.

## Results

The patients presented with hypermetabolic (n = 17; mean SUVmax: 11.6 ± 6.9) or hypometabolic (n = 14) brain lesions with different localizations, which are summarized in Table 1. Additionally, 13 patients had different additional metastatic sites with mean SUVmax: 9 ± 4 in different localizations (Table 2).

**Table 1.** **The summary of localizations of the brain lesions of the patients.**

**Number**	**Parietal**	**Occipital**	**Temporal**	**Frontal**	**Piriformis**	**Cavernosis**	**Cerebellum**	**Multiple**
n	11	8	6	5	1	1	3	5

**Table 2.** **The localizations of metastatic sites other than brain region.**

**Number**	**Lung**	**Cervical**	**Axilla**	**Mediastinal**	**Abdomen**	**Bone**	**Surrenal**	**Liver**
n	6	5	2	6	2	2	1	1

Three of the patients were documented to have benign brain disease despite positive MR consideration and were excluded from the analysis. In addition, two of the patients died during follow-up.

**Figure 1.**
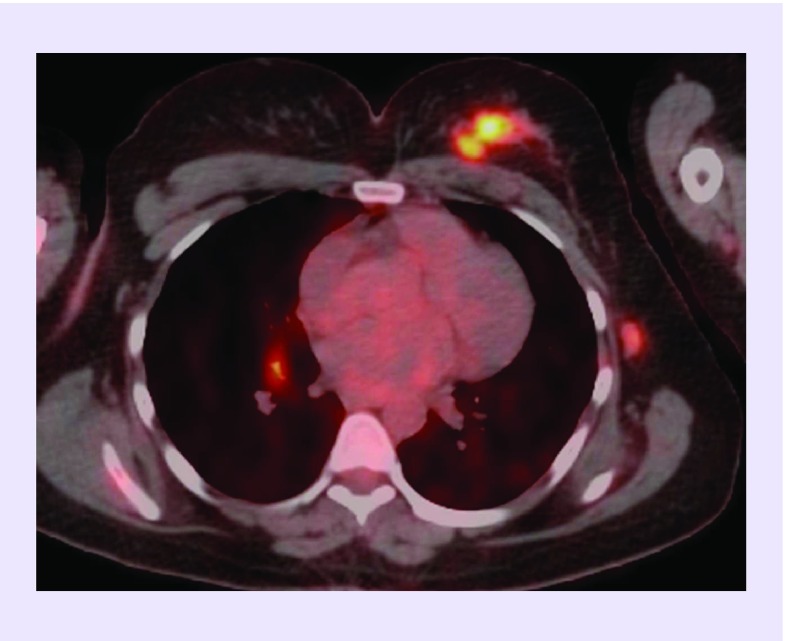
**Transaxial fused F-18 fluorodeoxyglucose PET/CT image of a patient with left breast primary tumor in diagnostic PET/CT whole body scan who has additional brain, bone and lymph node metastases.**

**Figure 2.**
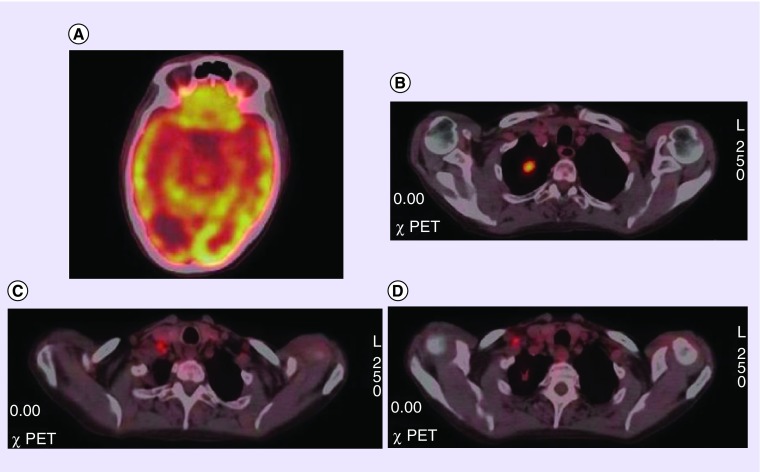
**F-18 F-18 fluorodeoxyglucose PET/CT image of a patient with hipometabolic brain lesion.** **(A)** F-18 fluorodeoxyglucose PET/CT axial images of brain region showing hypometabolic metastatic brain lesion. **(B)** Hypermetabolic lung lesion in the apex of right lung. **(C & D)** Lymph node metastasis in neck (level 6) and right supraclavicular region.

The primary tumor was determined by FDG PET/CT in 20/26 patients (77%) (lung [n = 6] (Figures 1 & 2), primary brain [n = 9], renal cell carcinoma [n = 2], skin [n = 1], breast [n = 1] (Figure 1) and neuroendocrine [n = 1]) (Table 3). Additionally, five patients were assumed to have primary lung (n = 3), nasopharyngeal (n = 1) and uterus (n = 1) carcinoma but histopathological results did not confirm the diagnosis. No primary tumor could be identified in one patient with squamous cell carcinoma metastasis. The other lesions were confirmed by histopathological results from their primary site and the brain lesions were also identified by biopsy results if they were benign.

**Table 3.** **The distribution of the primary tumors in the study group.**

**Diagnosis**	**Lung**	**Primary brain**	**Renal cell carcinoma**	**Skin**	**Breast**	**Neuroendocrine**
Number	n = 6	n = 9	n = 2	n = 1	n = 1	n = 1

## Discussion

The primary tumor with unknown metastasis is a problematic diagnosis and it is difficult and sometimes impossible despite careful diagnostic workup. The imaging modalities have certain limitations in this group but F-18 FDG PET/CT imaging has all the important advantages and superiorities in this group. First, whole body examination provides an overview of all possible sites of the body. Second, the high lesion/nonlesion ratio due to high affinity of most of the tumors to FDG provides easy detection of the primary tumor.

However, there are a limited number of studies in this group of patients and most of them involve all the cancer of unknown primary because this is not a frequent entity. Especially, the patients who presented with brain metastasis have not been evaluated in a large series except case reports. Jeong *et al*. evaluated 77 patients and concluded that FDG PET could be helpful in the determination of primary tumor [7]. It is important to divide these groups of patients in subgroups because the detection rates or estimations are very different in patients with liver or brain metastases. The detection rate of primary malignancy in the patients with brain metastases was higher than expected in our study group (77%). This result may be a consequence of improved image analysis methods or multislice CT integrated new-generation scanners. Additionally, increased number of patients with CUP resulted in an improved diagnostic power due to the experience in this field in recent years.

**Figure 3.**
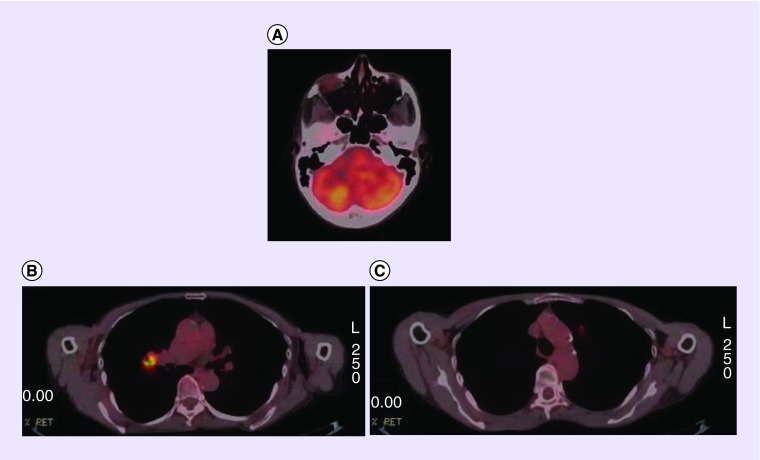
**F-18 F-18 fluorodeoxyglucose PET/CT images of a patient with hypermetabolic brain lesion.** **(A)** Axial projection of PET/CT images showing hypermetabolic brain metastasis in right cerebellar region. **(B & C)** Axial image of lung region showing right hilar hypermetabolic lesion and left metastatic pulmonary nodule.

In a retrospective analysis including 63 adult patients who presented with brain metastasis, the lung was the most common primary site (Figures 2 & 3) [8]. However, there were unexpected primary sites in several patients in this group, for example, breast cancer (Figure 1), skin cancer and renal cell cancer. The patients in this study were lung cancer as primary tumor besides primary brain tumor. A detailed review about CUP has highlighted that the patients with metastatic involvement of brain usually are adenocarcinoma or squamous cell carcinoma in origin, besides the primary site may not be determined in approximately 15% of these patients [9]. However, it is also documented that the patients with single brain lesions might benefit from surgery with better survival [10]. In a case report with single brain metastasis of adenocarcinoma in the patient, PET/CT successfully determined primary tumor in the left lung lobe which  was confirmed with histopathology result [11].

In a recent report with prognostic analysis of the patients with CUP patients in comparison with FDG PET/CT results concluded that PET/CT may also provide accurate estimation of prognosis based on the extent of disease even in case of lack of information of primary tumor [12]. A recent comparative study including the analysis of CUP with cervical metastases showed that FDG PET/CT is more sensitive than contrast-enhanced CT or MRI for detection of occult primary tumor [13]. Also, there may be non-FDG-avid lesions as a primary site in some patients whom different radiopharmaceuticals may be implicated [14]. As an example, one patient had neuroendocrine tumor metastasis in this study group; however, primary site was determined by PET/CT.

The treatment of these patients is another challenge with worse survival, as most of the patients do not have a favorable prognosis and life expectancy is short in these patients [15]. Two patients were dead in a short time interval after the PET/CT imaging in this study group. Thus, determination of the primary tumor and treatment in a timely manner are extremely important in this group of patients. PET/CT provides a single-stage estimation and determination of possible exampling site thereby providing quick analysis of the patients’ pathology.

Limitations of this study are its retrospective structure and a small sample size. However, a special group of patients was included in the study. Prospective studies in a large group of patients are warranted about this subject.

The detection rate in the patients with brain metastasis originating from a cancer of unknown primary is higher than previously reported in PET/CT. Thus, PET/CT must be performed in this group of patients in the first place in order to diagnose the patient in a timely manner.

## Future perspective

The important advances are ongoing in nuclear medicine thus new modalities may have a future role in the determination of the primary site of the disseminated cancer. One of these advances includes PET/MR device that is a promising tool, especially for brain imaging. PET/MR might contribute additional information with better spatial resolution, especially of soft tissues.
